# Childhood Maltreatment Was Correlated With the Decreased Cortical Function in Depressed Patients Under Social Stress in a Working Memory Task: A Pilot Study

**DOI:** 10.3389/fpsyt.2021.671574

**Published:** 2021-07-08

**Authors:** Mengying Ma, Xiao Zhang, Yuyanan Zhang, Yi Su, Hao Yan, Haoyang Tan, Dai Zhang, Weihua Yue

**Affiliations:** ^1^Institute of Mental Health, The Sixth Hospital, Peking University, Beijing, China; ^2^Key Laboratory of Mental Health, Ministry of Health & National Clinical Research Center for Mental Disorders, Peking University, Beijing, China; ^3^Lieber Institute for Brain Development, Baltimore, MD, United States; ^4^Department of Psychiatry and Behavioral Sciences, Johns Hopkins University School of Medicine, Baltimore, MD, United States; ^5^PKU-IDG/McGovern Institute for Brain Research, Peking University, Beijing, China

**Keywords:** stress, major depression disorder, working memory, anterior prefrontal cortex, childhood maltreatment

## Abstract

**Background:** Major depressive disorder (MDD) is a common psychiatric disorder associated with working memory (WM) impairment. Neuroimaging studies showed divergent results of the WM process in MDD patients. Stress could affect the occurrence and development of depression, in which childhood maltreatment played an important role.

**Methods:** Thirty-seven MDD patients and 54 healthy control subjects were enrolled and completed a WM functional magnetic resonance imaging task with maintenance and manipulation conditions under stress and non-stress settings. We collected demographical and clinical data, using 17-item Hamilton Depression Scale (HAMD-17) and Childhood Trauma Questionnaire (CTQ) in MDD patients. In the WM task, we analyzed the main diagnosis effect and explored the correlation of impaired brain regions in MDD patients with CTQ and HAMD-17.

**Results:** No group differences were found in the accuracy rate and reaction time between the two groups. MDD patients had lower brain activation in following regions (*P*_*FWE*_ < 0.05). The left fusiform gyrus showed less activation in all conditions. The right supplementary motor area (SMA) exhibited decreased activation under non-stress. The anterior prefrontal cortex showed reduced activation during manipulation under stress, with the β estimations of the peak voxel showing significant group difference negatively correlated with childhood sex abuse (*P*_Bonferroni_ < 0.05).

**Conclusions:** In our pilot study, MDD patients had reduced brain activation, affecting emotional stimuli processing function, executive function, and cognitive control function. Childhood maltreatment might affect brain function in MDD. This work might provide some information for future studies on MDD.

## Introduction

Major depressive disorder (MDD) is a common psychiatric disorder (1), which usually leads people to suffering from emotional disturbances and cognitive impairments (2, 3). Working memory (WM) involves the capability to memorize, retrieve, and utilize the information for a limited period (4) and is incredibly easily impaired in MDD patients (5, 6). Numbers of studies found widespread increased brain activations during the cognitive process in MDD patients (7), including the anterior prefrontal cortex (APFC) (8), dorsolateral prefrontal cortex (9, 10), and cingulate cortex (11). However, some studies showed hypoactive brain regions, including the frontal cortex, temporal cortex, insula, anterior cingulate cortex (ACC) and parietal cortex in depressed patients (12–14). These divergent results suggested the complexity of this issue, and potential factors might be the types of WM (15) and the levels of stress. Besides, childhood maltreatment has been considered to accelerate the development of depression (16–18). Moreover, a decreased volume of prefrontal cortex might play a mediated role in the relationship between childhood maltreatment and declined cognitive functioning (19–21). However, how can childhood maltreatment affect the WM process is complicated as stress has both direct neuroendocrine (22) and indirect methylation (23) effects on the development of depression. We hypothesized that the neural basis of the childhood maltreatment effects at different WM task conditions were different. To explore this hypothesis, we designed a WM task (24, 25) with varying subtasks (maintenance vs. manipulation) and varying stress levels (non-competition vs. competition) to compare the childhood maltreatment effects in different conditions.

## Materials and Methods

### Participants

In this study, 53 MDD patients and 64 healthy controls (HCs) were recruited. The patients were outpatients recruited from Peking University Sixth Hospital. We used the *Diagnostic and Statistical Manual of Mental Disorders, Fourth Edition, Text Revision* (*DSM-IV-TR*) diagnostic criteria of depression disorder, without other comorbidities of the *DSM-IV-TR* Axis I disorders. Two psychiatrists assessed the patients by using the Mini-International Neuropsychiatric Interview (version 5.0) (26). All HCs were enrolled by advertising in the community and social media and evaluated by using the Structured Clinical Interview for *DSM-IV-TR* Axis I Disorders, Research Version, Non-patient Edition to exclude any mental disorder (27). The current study was approved by the ethical committee of the Peking University Sixth Hospital. All the participants were given detailed information about the purpose and procedures of the study and signed the written consents.

We used the 17-item Hamilton Depression Scale (HAMD-17) to evaluate the symptom severity (28). In addition, we used Childhood Trauma Questionnaire (CTQ) to examine how could childhood maltreatment affect brain function in adult MDD patients (29). Subjects were included using the following criteria: (1) between 18 and 55 years of age, (2) right-handed, (3) Chinese Han lineage, and (4) MDD patients needed to get a HAMD-17 score ≥17. Subjects were excluded with the following criteria: (1) any current or history of neurological disease, (2) a history of more than 5-min loss of consciousness, (3) contraindications for magnetic resonance imaging (MRI) scanning, (4) electroconvulsive therapy within 6 months or history of severe medical illness, (5) other genetic disease, (6) serious impulsive behavior or suicide attempts, and (7) pregnancy and lactation.

We excluded subjects with low image quality or who did not complete the task (six MDD patients and four HCs) or with an accuracy rate of the maintenance <50% under competitive/non-competitive setting (two MDD patients, no HCs) or with head motion of more than 3° rotation/3-mm translation (eight MDD patients and six HCs). Finally, 37 MDD subjects and 54 HCs were included in the analysis (Table 1). Among the 37 patients, 10 patients were drug-naive. In addition, 17 MDD patients were taking selective serotonin reuptake inhibitors (fluoxetine, escitalopram, sertraline), six were taking serotonin norepinephrine reuptake inhibitors (venlafaxine, duloxetine), two were taking noradrenergic and specific serotonergic antidepressants (mirtazapine), one was taking dopamine norepinephrine reuptake inhibitors, DNRIs (bupropion), and one was taking flupentixol and melitracen.

**Table 1:** Demographic and Behavioral Characteristics of MDD patients and HCs.

**Characteristic**	**MDD patients (*n* = 37)**	**HCs (*n* = 54)**	***t*/χ^2^**	***p***
Age (years)	25.89 (4.75)	23.94 (3.05)	2.203	0.032
Gender (female/male)	23/14	29/25	0.641	0.423
Education (years)	16.54 (2.70)	16.72 (1.98)	−0.370	0.712
Duration of illness (months)	18.28 (29.92)			
HAMD-17 score	24.35 (5.60)			
CTQ total score	40.86 (10.89)			
Emotional abuse score of CTQ	8.72 (3.40)			
Physical abuse score of CTQ	6.31 (2.05)			
Sex abuse score of CTQ	5.92 (1.46)			
Emotional neglect score of CTQ	12.61 (5.11)			
Physical neglect score of CTQ	7.56 (3.02)			

*MDD, major depressive disorder; HCs, healthy controls; HAMD-17, 17-item Hamilton Depression Rating Scale; CTQ, Childhood Trauma Questionnaire*.

### WM Paradigm and Image Acquisition

**Figure 1:**
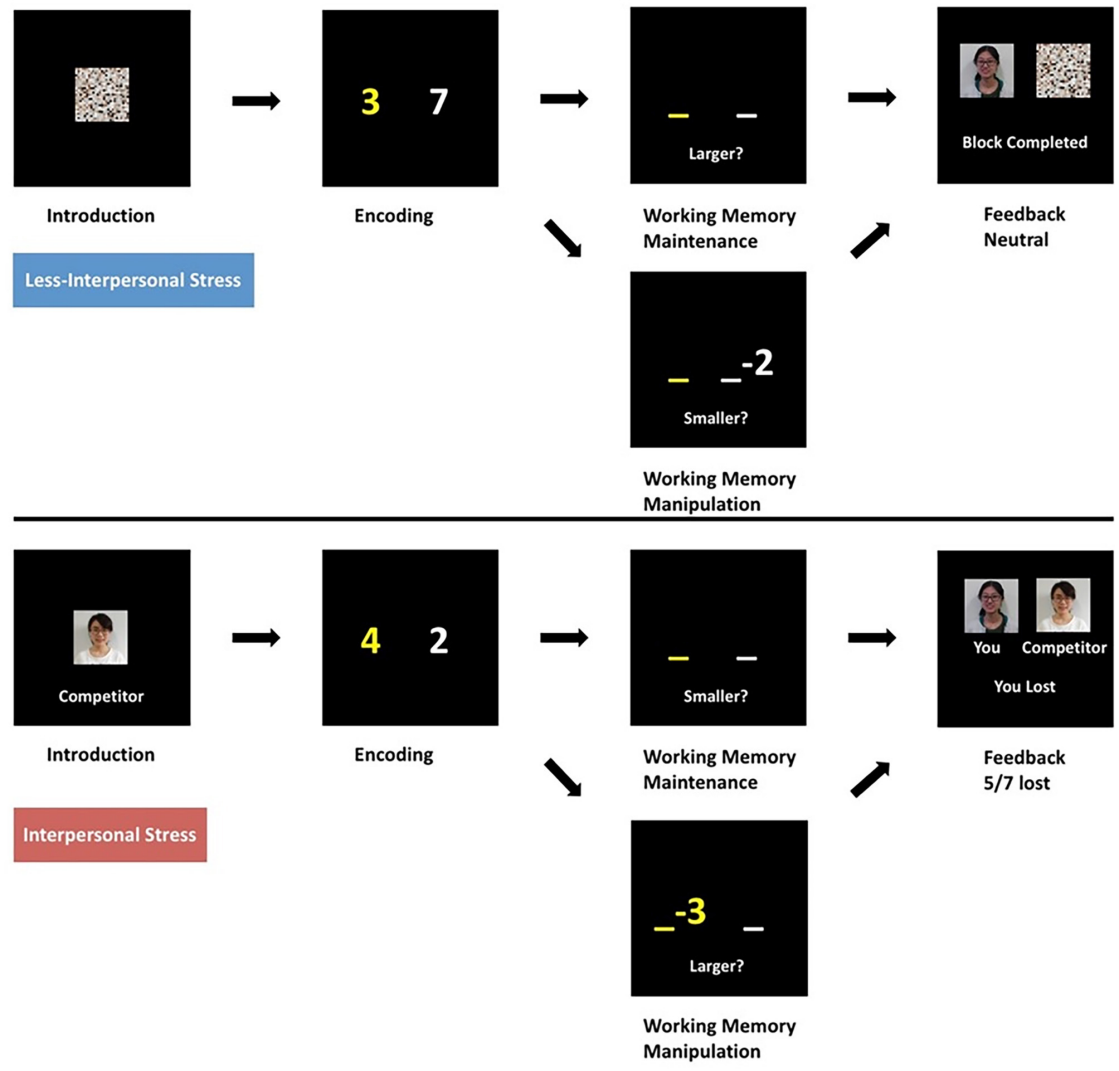
Working memory paradigm incorporating social competition stress. In the stressed component, subjects were led to believe that they were playing against a “competitor” of similar age and gender and were judged as winning or losing based on their speed and accuracy, which subsequently resulted in ~70% loss feedback. In the less stressed blocks, there was no competitor, and subjects received neutral feedback. In all the working memory manipulation and maintenance tasks, an array of two-number digits was encoded and held in working memory over 3 to 4 s. In working memory maintenance condition, subjects responded to which of the two maintained digits was larger or smaller as indicated. In working memory manipulation condition, subjects performed subtraction on one of the numbers held in working memory, followed by a response as to which result was larger or smaller as indicated. Subjects performed two runs counterbalanced for trial and stimuli presentation order over ~20 min. All instructions were translated to Chinese.

We developed an event-related “number calculation WM” task from previous works (30, 31) and newly comprised alternating competitive and non-competitive blocks (Figure 1). We validated that both the different subtasks and the different stress levels were successfully introduced in this task from our previous study (28), in which the detailed description of this task could be found. A 3.0-T GE Discovery MR750 scanner was used for scanning all participants at the Center for MRI Research, Peking University Institute of Mental Health. The parameters of the functional MRI are as follows: each echoplanar image included 33 (thickness/gap = 4.2/0 mm) axial slices, which covered the whole cerebrum and cerebellum (repetition time/echo time = 2,000/30 ms, flip angle = 90°, field of view = 22.4 × 22.4 cm^2^, matrix = 64 × 64). The protocol parameters were selected for optimizing the quality and stability of the blood oxygenation level-dependent signal with the exclusion of the first four images as dummy scans.

### Processing and Statistical Analyses of the MRI

We used MATLAB 2016b and SPM12 (http://www.fil.ion.ucl.ac.uk/spm) for analyzing the functional MRI data. The preprocessing of the data was performed as following steps: (1) slice timing correction, (2) realigning to the first volume and correcting the head motion, (3) spatially normalizing into standard stereotaxic space (Montreal Neurological Institute template) using a fourth-degree B-spline interpolation, and (4) using an 8-mm Gaussian kernel to spatial smoothing. After preprocessing, the voxel size of the image data was 3 × 3 × 3 mm^3^. We modeled every task-evoked stimulus as an independent delta function, and it convolved with the typical hemodynamic response function, controlling the systematic differences of global activity by normalizing ratio to the whole-brain global mean. And we used a 128-s high-pass filter for temporal filter. We modeled each event of task-evoked stimulus for performing trials correctly. In addition, we modeled the residual movement and incorrect response parameters as regressors of no interest. In this study, we planned to contrast the brain activation at the maintenance subtask or manipulation subtask under stress, non-stress setting and stress vs. non-stress, and between the two groups of MDD patients and HCs. Second-level analyses were subsequently taken, and the variability of intersubject was regarded as a random effect.

After controlling age, we used a flexible 2 × 2 analysis of variance in SPM12 to analyze the main effect of diagnosis, the main effect of stress, and the diagnosis × stress interaction effect. The significant level was set as *p* < 0.05 with whole-brain family-wise error (FWE) correction at both the maintenance subtask and the manipulation WM subtask. Then, we compared the main effect of diagnosis at stress maintenance, non-stress maintenance, stress manipulation, and non-stress manipulation separately in SPM12 to understand the group differences more specifically under each WM condition. The second-level analyses were carried on without any brain mask.

### Statistical Analyses of the Clinical and Behavioral Data

We used a standard statistical package (IBM SPSS 26.0, Chicago, IL) to analyze demographic and clinical data, including *t*-test and χ^2^ test. The behavioral data [accuracy rate and reaction time (RT)] of two groups at the maintenance or manipulation phase under stress or non-stress setting were analyzed by SPSS to explore the diagnosis × stress × task–difference interaction effect.

We first extracted the β estimations in the corresponding contrast images of each condition and setting in each group for the peak coordinates found in the aforementioned second-level brain image analyses. Then, we analyzed correlation between the β estimations values of the MDD group and the clinical variables (HAMD-17, CTQ). The level of statistical significance was set at *p* < 0.05 after Bonferroni correction for multiple comparisons.

## Results

### Demographic and Behavioral Results

We studied 37 MDD patients and 54 HCs who were currently living in Beijing. Both groups had similar gender distribution and had achieved similar educational levels. While HCs were slightly younger (Table 1). We included age as a covariate in subsequent analyses. MDD patients had an average illness duration of 18.28 months, with a mean HAMD-17 score of 24.35. We also obtained the CTQ for MDD patients, and the results are listed in Table 1.

**Figure 2:**
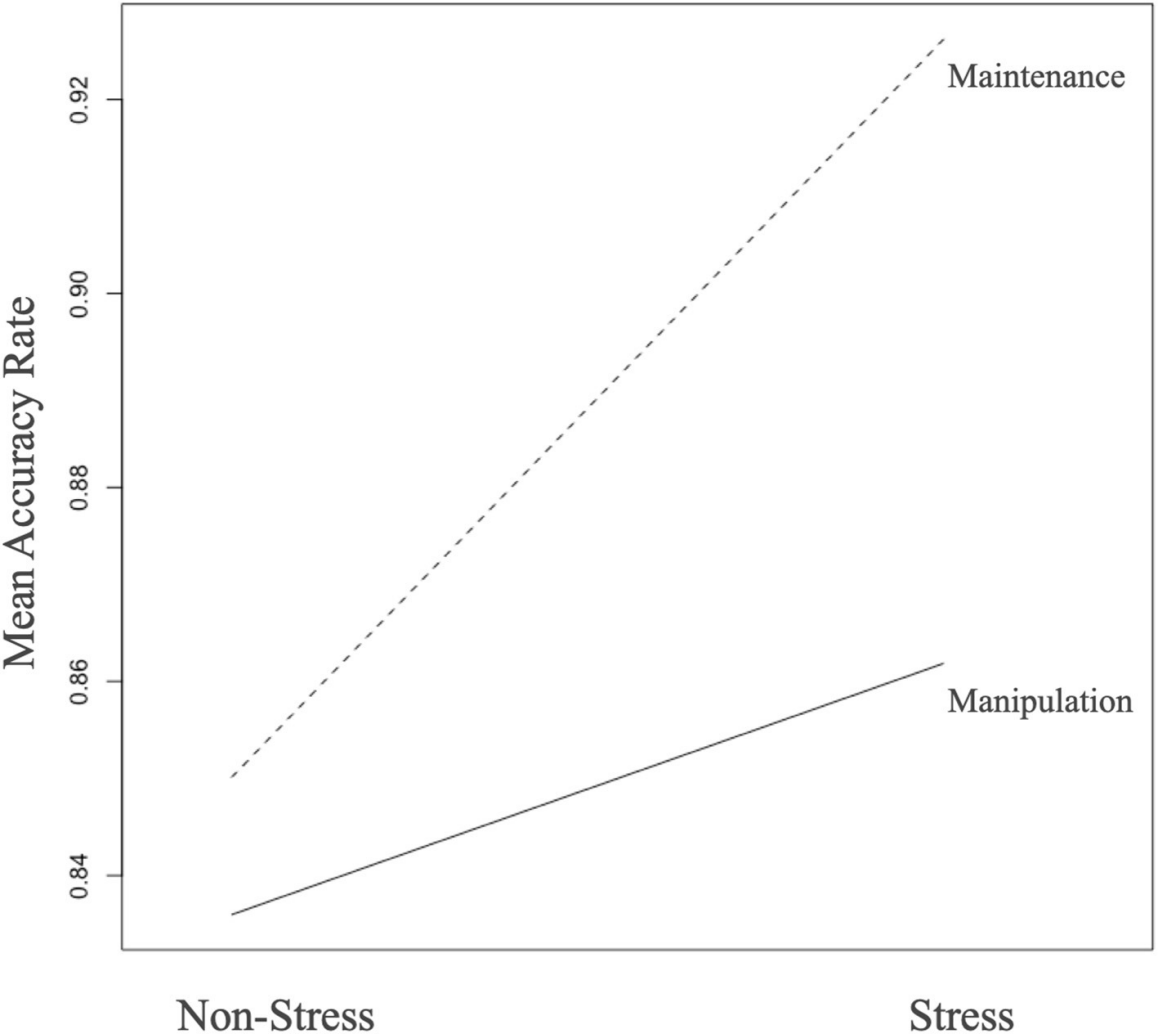
Stress and task difference in the whole sample (*n* = 91). During the WM maintenance condition, trials with stress were associated with relatively increased accuracy (*p* < 0.001). This effect was not so evident during the WM manipulation condition, resulting in a significant task by stress interaction (*p* = 0.002).

In terms of the accuracy rate, we observed a significant main effect of stress with a higher accuracy rate under the stress task (*F* = 30.586, *p* < 0.001), whereas, for task difference, we found a higher accuracy rate under maintenance task (*F* = 11.598, *p* < 0.001). The interaction effects of task difference × stress was significant (*F* = 10.126, *p* = 0.002; Figure 2). However, no group differences were found between MDD patients and HCs (Table 2). As for RT, we observed a significant main effect of task difference, with longer RT in the manipulation condition (*F* = 11.473, *p* = 0.001). Meanwhile, we observed a significant main effect of task-difference, with longer RT in the manipulation condition (*F* = 362.629, *p* < 0.001). There were no main effects of group, or interaction effect among the three factors.

**Table 2:** Description of behavioral performances of MDD patients and HCs.

	**MDD patients (*n* = 37)**	**HCs (*n* = 54)**	***t***	***p***
**Stress**				
Accuracy in WM maintenance	0.93 (0.06)	0.92 (0.08)	0.858	0.393
RT in WM maintenance (s)	1.16 (0.26)	1.18 (0.29)	−0.229	0.820
Accuracy in WM manipulation	0.86 (0.14)	0.86 (0.11)	0.189	0.850
RT in WM manipulation (s)	1.60 (0.37)	1.54 (0.37)	0.728	0.469
**Non-stress**				
Accuracy in WM maintenance	0.87 (0.08)	0.84 (0.09)	1.644	0.104
RT in WM maintenance (s)	1.24 (0.30)	1.22 (0.30)	0.214	0.831
Accuracy in WM manipulation	0.83 (0.16)	0.84 (0.12)	−0.225	0.823
RT in WM manipulation (s)	1.63 (0.37)	1.61 (0.33)	0.354	0.724

*MDD, major depressive disorder; HCs, healthy controls; RT, reaction time; WM, working memory*.

### WM-Related Brain Activation

**Figure 3:**
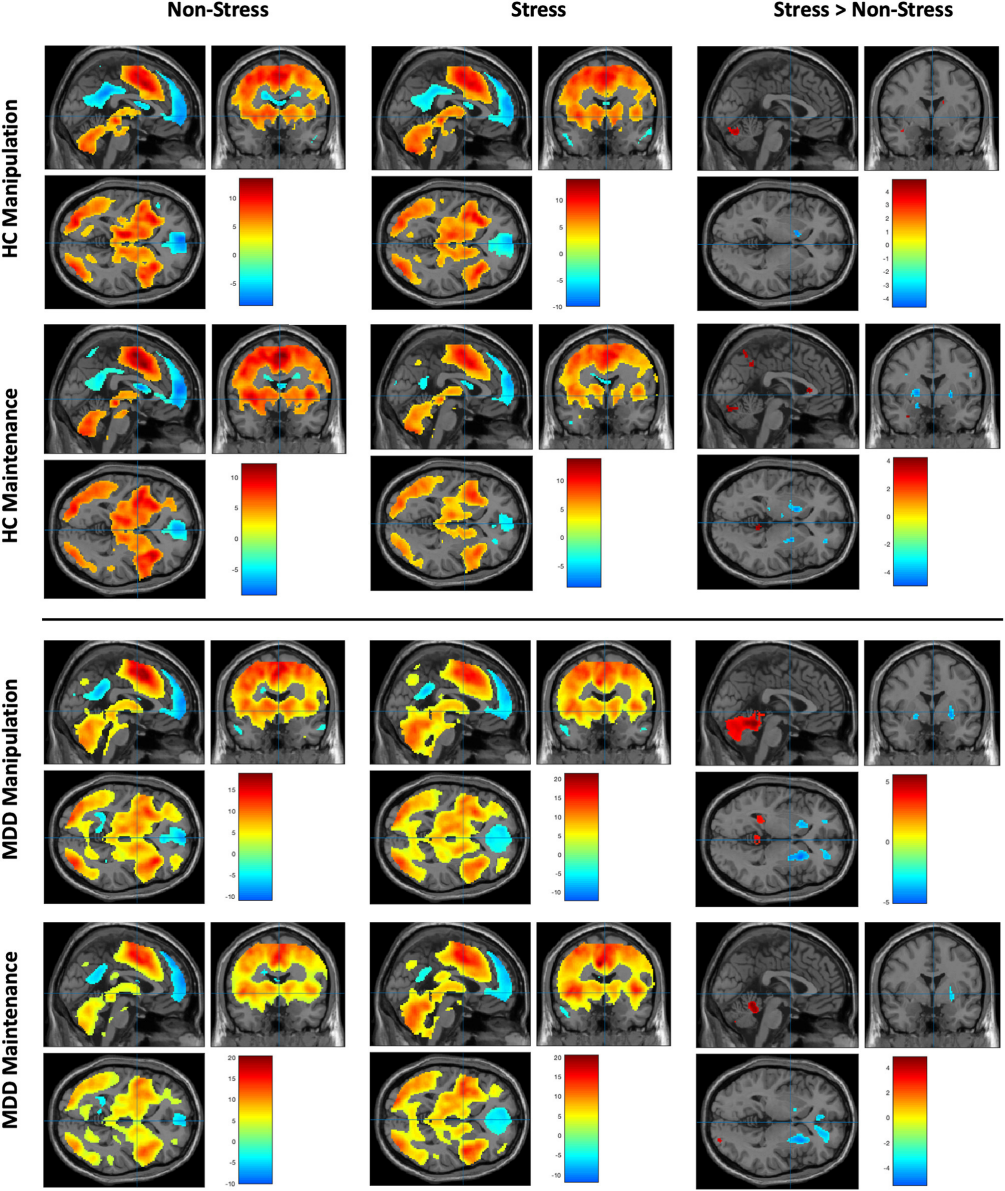
Working memory–related brain activity and stress effect in MDD patients and healthy control subjects (data were shown for *p* < 0.001, uncorrected). Upper left: The brain activity of WM manipulation and maintenance subtasks in MDD group under non-stress setting. Upper middle: The brain activity of WM manipulation and maintenance subtasks in MDD group under stress setting. Upper right: The brain activity of comparison of stress vs. non-stress setting within the MDD group under different subtask patterns. Bottom left: The brain activity of WM manipulation and maintenance subtasks in HC group under non-stress setting. Bottom middle: The brain activity of WM manipulation and maintenance subtasks in HC group under stress setting. Bottom right: The brain activity of comparison of stress vs. non-stress setting within the HC group under different subtask patterns.

During each of the WM maintenance and manipulation conditions under stress or non-stress setting in both MDD and HC groups, regions in the prefrontal, parietal, temporal, occipital cortices, and striatum were robustly activated, along with well-established deactivation in areas of default mode network during cognitive task, including the medial PFC (MPFC) and posterior cingulate cortex (*p* < 0.05, whole-brain FWE correction; Figure 3, Supplementary Tables 1, 2).

### Group Differences Under WM Maintenance Condition

Under the WM maintenance condition, the main effect of stress and interaction effect of diagnosis × stress were not significant. However, the main effect of diagnosis was significant in the left fusiform, left postcentral gyrus, middle cingulum, left superior temporal gyrus, and left precuneus (*p* < 0.05 whole-brain FWE correction, cluster >50; Table 3).

**Table 3:** Main effect of group difference between MDD patients and HCs (controlling for age, *p* < 0.05, voxel-wise whole-brain FWE corrected, cluster size >50).

**Peak Region**	**Cluster**	***x***	***y***	***z***	***F* score**
**Maintenance**					
L fusiform gyrus	225	−50	−60	−16	54.45
L postcentral gyrus	50	−52	−28	56	45.61
Middle cingulum	81	0	8	40	35.13
L superior temporal gyrus	60	−58	6	2	30.92
L precuneus	87	−12	−78	52	29.59
**Manipulation**					
R superior frontal gyrus	76	4	16	62	56.58
L fusiform gyrus	291	−50	−60	−16	50.41
L precuneus	151	−10	−76	54	43.55
R middle occipital gyrus	77	28	−94	−6	37.37
L middle frontal gyrus	135	−46	6	50	34.43
L cingulate gyrus	66	−2	8	40	34.06
L inferior occipital gyrus	87	−40	−86	−6	33.71
R superior frontal gyrus	149	30	56	−4	32.65
R middle frontal gyrus	59	44	30	42	31.97
R middle frontal gyrus	58	32	0	64	31.77
L culmen	72	−34	−42	−30	30.97
R pyramis	52	26	−70	−42	26.45

*MDD, major depressive disorder; HCs, healthy controls, L, left; R, right*.

**Figure 4:**
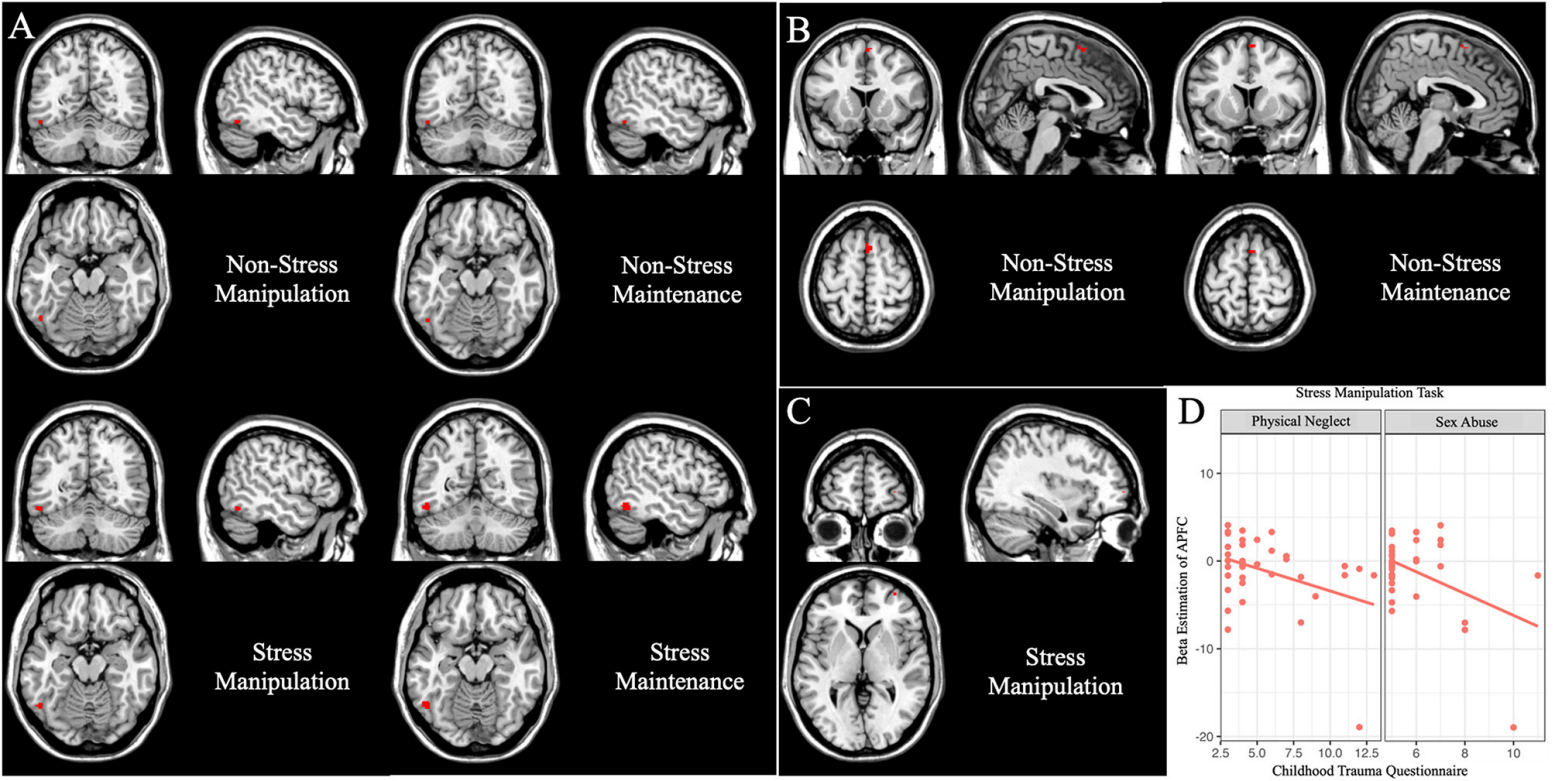
Group differences under working memory maintenance and manipulation subtasks (data were controlling for age, *p* < 0.05, voxel-wise whole-brain FWE corrected). Main group differences under the working memory maintenance subtask. **(A)** The left fusiform gyrus showed decreased activation in MDD patients under the maintenance and manipulation conditions in stress and non-stress settings. **(B)** The right SMA exhibited reduced activation in MDD patients under the maintenance and manipulation conditions only in non-stress setting. **(C)** Under stress setting, MDD patients showed less activation in the right APFC in the manipulation condition. **(D)** In MDD group, and the β estimations of the right APFC peak voxel under stress manipulation subtask were negatively correlated with sex abuse (*r* = −0.43, *p* = 0.008) and physical neglect (*r* = −0.37, *p* = 0.026) score in the Childhood Trauma Questionnaire.

Then, we focused on the group differences under stress setting (*p* < 0.05, whole-brain FWE correction, cluster >2; Figure 4). MDD patients had reduced activation in the left fusiform (*x* = −50, *y* = −60, *z* = −14, *T* = 5.60, cluster size = 49). While under non-stress setting (*p* < 0.05 whole-brain FWE correction, cluster > 2; Figure 4), MDD patients had decreased activation in the right supplementary motor area (SMA) (*x* = 4, *y* = 14, z = 62, *T* = 5.60, cluster size = 13) and left fusiform gyrus (*x* = −50, *y* = −60, *z* = −16, *T* = 4.92, cluster size = 6).

### Group Differences Under WM Manipulation Condition

Under the WM manipulation condition, the main effect of stress and interaction effect of diagnosis × stress were not significant. However, the main effect of diagnosis was significant in many brain regions, including the fusiform gyrus, precuneus, cingulate gyrus, inferior occipital gyrus, culmen in the left hemisphere, and superior frontal gyrus, middle occipital gyrus, superior frontal gyrus, middle frontal gyrus, and pyramis in the right hemisphere (*p* < 0.05 FWE correction, cluster >50; Table 3).

Then, we focused on the group differences under stress setting (*p* < 0.05, whole-brain FWE correction, cluster > 2; Figure 4), MDD patients showed less activation in the left fusiform (*x* = −52, *y* = −60, *z* = −16, *T* = 5.12, cluster size = 14), and right APFC (*x* = 32 *y* = 60, *z* = 2, *T* = 4.93, cluster size = 3). While under non-stress setting, the main effect of diagnosis (*p* < 0.05 whole-brain FWE correction) lay in the right SMA (*x* = 4, *y* = 14, *z* = 62, *T* = 5.75, cluster size = 23) and left fusiform gyrus (*x* = −50, *y* = −60, *z* = −16, *T* = 4.97, cluster size = 7).

### Correlation Analysis

In the MDD patients, we did not find any significant correlation between the HAMD-17 score and the β estimations of the peak voxel, which showed significant difference between HCs and MDD patients. While we found the β estimations of the peak voxel in APFC under stress manipulation task (*x* = 32, *y* = 60, *z* = 2) were negatively correlated with CTQ sex abuse (*r* = −0.43, *p* = 0.008; Figure 4D). These β estimations were also negatively correlated with CTQ physical neglect (*r* = −0.37, *p* = 0.026; Figure 4D), but could not withstand Bonferroni correction.

## Discussion

### Main Findings

In the current pilot study, we aimed to explore the neural changes in MDD patients by investigating brain function associated with a stress-related WM task. We found that compared to HCs, MDD patients showed comprehensive less brain activation during both the WM maintenance and manipulation conditions. Particularly, we found decreased brain activation in the left fusiform under both stress and non-stress settings in both WM maintenance and manipulation conditions. Notably, the activation in the right SMA showed group differences in both WM maintenance and manipulation conditions under non-stress but not stress setting. We also found a reduced APFC activation in MDD under WM manipulation task under stress setting, which was negatively correlated with the CTQ sex abuse.

### Left Fusiform Gyrus

Our study found that the activation of the left fusiform gyrus was decreased in MDD patients in both maintenance and manipulation conditions under non-stress or stress setting. The fusiform gyrus is the most crucial part of the high-level visual cortex, which is associated with the recognition of facial expressions (32, 33), integration of cognitive information, and emotional modulation (34). Previous studies had observed reduced gray matter volume, thinner cortical thickness, and less surface area of the fusiform gyrus in patients with depression (35–37). The fusiform gyrus may integrate the emotional and cognitive processes by modulating the visual stimulation processes. Moreover, compared to HCs, the connectivity between the fusiform gyrus and medial orbitofrontal cortex was decreased in MDD patients (38), which suggested that the fusiform gyrus had an indirect effect on the WM function and emotional modulation. In addition, the reduced activation of fusiform gyrus may also be related to the impaired function of the attention biases of negatively emotional stimuli (32, 39). We speculated that in the WM task, the role of the emotional stimuli process was impaired under both stress and non-stress settings or whether patients with MDD saw the face of a competitor or not.

### Right Supplementary Motor Area

Meta-analyses showed consistent activation of SMA in the WM task, which indicated that the SMA included in the widespread frontoparietal network was part of the core WM network (4, 40, 41). Besides, the visual attention function of executive function is regulated by the SMA, precentral gyrus and ACC cortical network (42), which benefits the linking of sensory information to the learning and execution of movement sequences (43). Reduced SMA volumes and impairment in implicit motor sequence learning have been observed in MDD patients (44). Meanwhile, Sarkheil et al. (45) found that the SMA was associated with the psychomotor features (such as motor behavior) of depression, and they speculated that the increased functional connectivity between the SMA and other regions might suggest that recruiting more brain resources was needed for completing the more complicated task in MDD patients. Moreover, MDD patients were sensitive to stress (46). In our study, the decreased activation of SMA under non-stress but not stress setting might also support this view. As a negative stimulation, the face of a competitor might affect the visual attention of executive function, which resulted in “they potentially required additional serial mental processing steps” (47). Hence, the function of the SMA in MDD patients might be increased for making compensation in the WM task under stress, which led to increasing the activation of the SMA and reducing the difference compared to HCs.

### Anterior Prefrontal Cortex

Studies showed that the APFC (Brodmann area 10, BA10) was involved in WM, episodic memory, prospective memory, and the consideration of multiple relations in the meantime (48, 49). Compared to children with non-depressed mothers, children with depressed mothers showed decreased activation in the APFC during the N-back task (50), which suggested that the activation of APFC might be an endophenotype of depression. Besides, the activation of the APFC has been observed in relatively simple tasks and would increase with the difficulty load of WM task in healthy subjects (51). Moreover, a prior work reported that higher activation in the APFC was related to WM and choice-difficulty effects associated with self-control (52). Besides, APFC is a brain region that is sensitive to stress (53), and subjects with posttrauma stress disorder showed decreased gray matter volume in APFC compared with control subjects (54). Hence, MDD patients might keep the same cognitive control function with HCs under the non-stress setting, or in the maintenance condition in the stress setting. However, facing both stress and manipulation subtask, which means more choice difficulties, the cognitive control function in MDD patients was decreased compared to HCs.

### Childhood Sex Abuse and APFC Activation

Childhood maltreatment, symptoms of negative emotionality, poor friend support, and externalizing problems in childhood and adolescence are risk factors for early-onset MDD patients (55, 56). Besides, childhood maltreatment can cause a series of physiological and neurohumoral reactions, including reduced volumes in the prefrontal cortex (57), and may lead individuals to being susceptible to depression (58–60). Previous studies exhibited that the volumes of ventromedial PFC and rostral prefrontal cortex were reduced in children and adults who suffered from physical and sexual abuse (61, 62). Furthermore, compared to individuals without childhood maltreatment, the activity in the MPFC was decreased in individuals who suffered from childhood maltreatment during emotional and neutral memory encoding and recognition (22). Meanwhile, substance abuse and stress can bring about long-lasting changes by modulating of gene expression or epigenetic mechanisms in the brain, and indeed an abnormal pattern of genome-wide DNA methylation in APFC of subjects with alcohol use disorder (63). In our study, childhood sex abuse might disrupt the function of APFC in direct and indirect ways, such as brain activation and DNA methylation, and then contribute to the development of depression.

## Limitations

There are several potential limitations in our study. First, the sample size was not large enough. Therefore, it was only a pilot study. In the future, we need to enlarge the sample to test and verify the current findings. Second, we did not distinguish the depression subtypes, which might be the reason why there was no correlation between the β estimations of peak voxel and the HAMD-17 score. Therefore, we need to investigate the differences among different subtypes of depression and study the relationship between clinical symptoms and underline mechanism in a larger sample.

## Conclusion

In our pilot study, the decreased brain activation of the left fusiform gyrus, SMA, and APFC helps us to understand the abnormalities of the emotional stimuli processing function, executive function, and cognitive control function in MDD. Childhood maltreatment might play a crucial role in the development of MDD. Although, the findings of this study might not be conclusive, they could provide some information for other researchers. In the future, we need to explore the impaired brain circuits under stress, including the function and connection between the brain regions, which were found in our article in a larger sample.

## Data Availability Statement

The data supporting the conclusions of this article will be made available by the authors, without undue reservation.

## Ethics Statement

The studies involving human participants were reviewed and approved by the Ethical Committee of the Peking University Sixth Hospital. The patients/participants provided their written informed consent to participate in this study.

## Author Contributions

WY and DZ designed and supervised the study. HT designed the task. MM, YZ, XZ, and YS recruited subjects and performed the study. MM, YZ, and XZ organized data. MM and XZ analyzed the data and wrote the paper. HY, HT, and WY gave instruction for the analysis and modified the paper. All collaborators reviewed and approved the final manuscript.

## Conflict of Interest

The authors declare that the research was conducted in the absence of any commercial or financial relationships that could be construed as a potential conflict of interest.
