# Supplementary material for: Childhood Maltreatment Was Correlated With the Decreased Cortical Function in Depressed Patients Under Social Stress in a Working Memory Task: A Pilot Study
Source: Front Psychiatry. 2021 Jul 8;12:671574. doi: 10.3389/fpsyt.2021.671574 (PMC8295536; doi:10.3389/fpsyt.2021.671574)
Supplement: Supplementary file 1 [file Table_1.DOCX]

Supplementary Materials

Supplementary Tables:

Table S1. Brain activation in healthy controls during working memory maintenance and manipulation under stress or non-stress settings (controlling for age, p< 0.05, voxel-wise whole-brain FWE corrected, cluster size > 10).

| Peak Region | Cluster | x | y | z | T score |
| --- | --- | --- | --- | --- | --- |
| **Manipulation: Stress** | | | | | |
| L Postcentral Gyrus | 105527 | -52 | -22 | 48 | 21.32 |
| L Postcentral Gyrus |  | -38 | -24 | 52 | 18.96 |
| L Precentral Gyrus |  | -32 | -14 | 62 | 18.28 |
| R Middle Cingulum | 41 | 16 | -28 | 42 | 5.96 |
| L Angular Gyrus | 316 | -48 | -74 | 32 | 12.06 |
| L Angular Gyrus |  | -56 | -60 | 38 | 5.78 |
| L Precuneus | 3524 | -6 | 46 | 50 | 11.25 |
| R Superior Medial Frontal Gyrus |  | 6 | 54 | 40 | 10.71 |
| R Superior Medial Frontal Gyrus |  | 12 | 44 | 52 | 10.18 |
| R Posterior Cingulum Gyrus | 560 | 2 | -50 | 28 | 8.38 |
| L Precuneus |  | -10 | -56 | 10 | 7.64 |
| R Precuneus |  | 10 | -52 | 12 | 6.76 |
| R Hippocampus | 62 | 26 | -8 | -22 | 8.36 |
| R Middle Temporals Gyrus | 285 | 56 | -62 | 22 | 7.99 |
| R Angular Gyrus |  | 52 | -68 | 32 | 7.44 |
| L Orbital Middle Frontal Gyrus | 27 | -28 | 40 | -12 | 6.16 |
| L Orbital Inferior Frontal Gyrus |  | -36 | 34 | -16 | 5.43 |
| R Middle Temporal Pole | 12 | 40 | 16 | -38 | 5.74 |
|  | | | | | |
| **Manipulation: Non-Stress** | | | | | |
| L Inferior Parietal Lobule | 67769 | -52 | -26 | 44 | 18.73 |
| L Postcentral Gyrus |  | -42 | -32 | 44 | 18.32 |
| L Inferior Parietal Lobule |  | -32 | -54 | 44 | 18.32 |
| R Superior Temporal Gyrus | 31 | 62 | -32 | 18 | 5.94 |
| R Superior Frontal Gyrus | 2765 | 18 | 38 | 54 | 10.75 |
| R Superior Medial Frontal Gyrus |  | 4 | 66 | 16 | 10.65 |
| L Superior Medial Frontal Gyrus |  | -8 | 46 | 50 | 9.78 |
| L Angular Gyrus | 289 | -46 | -76 | 32 | 10.56 |
| L Angular Gyrus |  | -56 | -60 | 38 | 6.00 |
| L Precuneus | 1401 | -10 | -56 | 10 | 9.39 |
| R Precuneus |  | 10 | -54 | 14 | 8.27 |
| L Posterior Cingulum Gyrus |  | 0 | -50 | 28 | 8.02 |
| R Angular Gyrus | 271 | 52 | -66 | 30 | 8.51 |
| R Anterior Cingulum Gyrus | 17 | 2 | 38 | 0 | 6.01 |
|  |  |  |  |  |  |
| **Manipulation: Stress > Non-Stress** | | | | | |
| Vermis 4-5 | 73 | 2 | -46 | -14 | 5.51 |
|  | | | | | |
| **Maintenance: Stress** | | | | | |
| L Middle Cingulum | 73688 | -6 | 2 | 40 | 20.34 |
| L Inferior Parietal Lobule |  | -52 | -26 | 46 | 19.96 |
| L Postcentral Gyrus |  | -40 | -30 | 46 | 18.94 |
| R Middle Temporals Gyrus | 142 | 52 | -22 | -10 | 6.90 |
| L Olfactory | 17 | -22 | 4 | -18 | 5.93 |
| R Superior Medial Frontal Gyrus | 3529 | 10 | 44 | 52 | 11.80 |
| L Superior Medial Frontal Gyrus |  | -6 | 46 | 50 | 11.42 |
| R Superior Medial Frontal Gyrus |  | 8 | 52 | 42 | 10.92 |
| L Angular Gyrus | 220 | -48 | -74 | 32 | 10.04 |
| L Angular Gyrus |  | -52 | -66 | 38 | 5.22 |
| R Hippocampus | 49 | 26 | -8 | -22 | 7.51 |
| R Middle Temporals Gyrus | 213 | 56 | -62 | 22 | 7.50 |
| R Angular Gyrus |  | 52 | -66 | 28 | 7.01 |
| L Precuneus | 49 | -10 | -56 | 10 | 6.56 |
| L Orbital Inferior Frontal Gyrus | 37 | -36 | 34 | -16 | 6.53 |
| L Orbital Middle Frontal Gyrus |  | -28 | 38 | 14 | 5.75 |
| R Orbital Middle Frontal Gyrus | 41 | 28 | 40 | -12 | 6.15 |
|  | | | | | |
| **Maintenance: Non-Stress** | | | | | |
| L Inferior Parietal Lobule | 61629 | -52 | -24 | 48 | 20.19 |
| L Inferior Parietal Lobule |  | -42 | -30 | 42 | 17.55 |
| L Precentral Gyrus |  | -34 | -16 | 60 | 17.27 |
| R Middle Temporals Gyrus | 109 | 50 | -22 | -12 | 7.4 |
| R Middle Cingulum | 56 | 14 | -28 | 42 | 6.18 |
| R Lingual Gryus | 50 | 24 | -58 | -2 | 5.33 |
| R Calcarine |  | 18 | -66 | -2 | 5.29 |
| R Superior Medial Frontal Gyrus | 2183 | 10 | 42 | 54 | 9.96 |
| R Superior Medial Frontal Gyrus |  | 6 | 68 | 14 | 9.69 |
| L Superior Medial Frontal Gyrus |  | -10 | 44 | 52 | 9.63 |
| L Angular Gyrus | 155 | -46 | -76 | 32 | 8.02 |
| L Precuneus | 460 | -8 | -58 | 12 | 7.99 |
| R Precuneus |  | 8 | -54 | 18 | 6.17 |
| L Posterior Cingulum Gyrus |  | 0 | -50 | 30 | 5.86 |
| R Middle Occipital Gyrus | 126 | 52 | -68 | 26 | 6.34 |
| L ParaHippocampal Gyrus | 14 | -34 | -40 | -10 | 5.51 |

Table S2. Brain activation in MDD patients during working memory maintenance and manipulation under stress or non-stress settings (controlling for age, p< 0.05, voxel-wise whole-brain FWE corrected, cluster size > 10).

| Peak Region | Cluster | x | y | z | T score |
| --- | --- | --- | --- | --- | --- |
| **Manipulation: Stress** | | | | | |
| L Precentral Gyrus | 40141 | -36 | -12 | 58 | 13.88 |
| L Inferior Parietal Gyrus |  | -36 | -40 | 44 | 12.84 |
| L Postcentral Gyrus |  | -40 | -32 | 54 | 11.69 |
| R Operculum Inferior Frontal Gyrus | 2001 | 54 | 10 | 10 | 10.10 |
| R Insula |  | 38 | 18 | 0 | 9.83 |
| R Operculum Inferior Frontal Gyrus |  | 60 | 12 | 22 | 8.85 |
| R Middle Frontal Gyrus | 199 | 32 | 40 | 26 | 6.92 |
| R Middle Frontal Gyrus |  | 38 | 34 | 28 | 5.50 |
| L Superior Medial Frontal Gyrus | 2953 | -6 | 42 | 52 | 9.83 |
| R Superior Frontal Gyrus |  | 18 | 40 | 52 | 9.66 |
| R Superior Medial Frontal Gyrus |  | 12 | 52 | 32 | 8.41 |
| L Angular Gyrus | 491 | -52 | -66 | 26 | 9.46 |
| R Orbital Middle Frontal Gyrus | 90 | 34 | 40 | -12 | 8.41 |
| R Angular Gyrus | 254 | 50 | -64 | 34 | 7.97 |
| L Middle Cingulum | 486 | 0 | -40 | 36 | 6.68 |
| L Precuneus |  | -4 | -62 | 20 | 6.64 |
| R Precuneus |  | 6 | -52 | 26 | 6.61 |
|  | | | | | |
| **Manipulation: Non-Stress** | | | | | |
| L Inferior Parietal Lobule | 26826 | -36 | -38 | 44 | 13.37 |
| L Precentral Gyrus |  | -40 | -8 | 54 | 13.28 |
| L Precentral Gyrus |  | -36 | -14 | 58 | 7.75 |
| R Superior Cerebelum | 8913 | 34 | -52 | -36 | 12.32 |
| R Superior Parietal Lobule |  | 26 | -62 | 50 | 10.12 |
| R Orbital Inferior Frontal Gyrus | 2104 | 34 | 22 | -10 | 9.41 |
| R Insula |  | 46 | 6 | 2 | 9.11 |
| R Middle Frontal Gyrus | 168 | 30 | 34 | 26 | 7.17 |
| R Middle Frontal Gyrus |  | 42 | 34 | 28 | 5.20 |
| L Superior Medial Frontal Gyrus | 2469 | -6 | 60 | 2 | 8.85 |
| R Anterior Cingulum Gyrus |  | 8 | 52 | 14 | 8.77 |
| R Superior Frontal Gyrus |  | 20 | 42 | 50 | 8.15 |
| L Angular Gyrus | 412 | -46 | -72 | 26 | 7.93 |
| L Angular Gyrus |  | -52 | -68 | 36 | 7.51 |
| L Posterior Cingulum Gyrus | 735 | 0 | -44 | 32 | 7.72 |
| L Calcarine |  | -6 | -64 | 20 | 5.11 |
| R Posterior Cingulum Gyrus |  | 6 | -48 | 28 | 4.79 |
| R Angular Gyrus | 133 | 52 | -66 | 30 | 5.60 |
|  | | | | | |
| **Maintenance: Stress** | | | | | |
| L Precentral Gyrus | 30073 | -36 | -12 | 58 | 13.77 |
| L Middle Cingulum |  | -2 | 10 | 42 | 11.52 |
| L Precentral Gyrus |  | -34 | -24 | 62 | 11.31 |
| R Operculum Inferior Frontal Gyrus | 1662 | 56 | 12 | 8 | 8.95 |
| R Insula |  | 42 | 4 | 6 | 8.70 |
| R Insula |  | 34 | 20 | 2 | 8.26 |
| L Thalamus | 430 | -12 | -16 | 2 | 7.58 |
| R Thalamus |  | 6 | -18 | 6 | 6.21 |
| L ParaHippocampal Gyrus | 36 | -20 | -30 | -14 | 6.67 |
| R Middle Frontal Gyrus | 65 | 34 | 40 | 26 | 6.58 |
| R Caudate | 96 | 10 | 10 | -6 | 6.33 |
| L Middle Frontal Gyrus | 19 | -30 | 52 | 14 | 5.91 |
| R Superior Frontal Gyrus | 871 | 16 | 44 | 50 | 8.62 |
| L Superior Medial Frontal Gyrus |  | -10 | 46 | 50 | 7.79 |
| L Superior Medial Frontal Gyrus |  | -6 | 60 | 12 | 7.54 |
| R Orbital Middle Frontal Gyrus | 35 | 32 | 38 | -14 | 7.13 |
| L Angular Gyrus | 32 | -50 | -72 | 32 | 6.00 |
| R Angular Gyrus | 24 | 52 | -68 | 30 | 5.98 |
|  | | | | | |
| **Maintenance: Non-Stress** | | | | | |
| L Superior Frontal Gyrus | 23348 | -24 | -8 | 62 | 12.07 |
| R Supplementary Motor Area |  | 2 | -2 | 58 | 11.94 |
| L Precentral Gyrus |  | -36 | -14 | 58 | 11.94 |
| R Operculum Inferior Frontal Gyrus | 2507 | 58 | 12 | 8 | 10.73 |
| R Insula |  | 32 | 22 | -2 | 10.13 |
| R Precentral Gyrus |  | 52 | 8 | 30 | 9.42 |
| R Cerebelum 6 | 3268 | 34 | -52 | -34 | 10.47 |
| R Superior Parietal Lobule | 3761 | 18 | -68 | 52 | 10.46 |
| R Postcentral Gyrus |  | 50 | -28 | 42 | 9.58 |
| R Angular Gyrus |  | 28 | -54 | 46 | 8.92 |
| R Caudate | 181 | 8 | 6 | 4 | 6.35 |
| R Pallidum |  | 18 | 4 | 6 | 6.04 |
| R Middle Frontal Gyrus | 83 | 32 | 34 | 26 | 6.60 |
| L Insula | 77 | -32 | -18 | 4 | 6.29 |
| R Superior Medial Frontal Gyrus | 2389 | 4 | 54 | 14 | 9.01 |
| R Superior Medial Frontal Gyrus |  | 4 | 62 | 16 | 8.87 |
| L Superior Medial Frontal Gyrus |  | -10 | 46 | 50 | 8.44 |
| L Middle Occipital Lobule | 183 | -46 | -78 | 28 | 7.98 |
| L Angular Gyrus |  | -52 | -68 | 24 | 6.82 |
| L Middle Occipital Lobule |  | -40 | -80 | 34 | 6.66 |
| R Angular Gyrus | 19 | 54 | -62 | 28 | 5.74 |
